# A study of management decisions to adopt emission reduction measures in heavy industry in an emerging economy

**DOI:** 10.1038/s41598-023-28417-2

**Published:** 2023-01-25

**Authors:** Togar Wiliater Soaloon Panjaitan, Paul Dargusch, David Wadley, Ammar Abdul Aziz

**Affiliations:** 1 School of Earth and Environmental Sciences, University of Queensland, Brisbane, 4072 Australia; 2 Industrial Engineering Department, Petra Christian University, Surabaya, Indonesia; 3 Department of Geography, Universitas Negeri Malang, East Java, 65145 Indonesia; 4 School of Agriculture and Food Sciences, University of Queensland, Gatton, 4343 Australia

**Keywords:** Climate-change policy, Sustainability

## Abstract

Heavy industry can face challenges in choosing applicable climate change mitigation measures due to a lack of technical background and practical guidance. A better understanding of these determinants is needed to design region-specific climate policies that most effectively enable more ‘successful’ low carbon transitions. Set in an emerging economy, this study aims to understand the determinants which underlie investment decision-making in greenhouse gas reduction. It relies on empirical research using an exploratory case study method in the leading cement company in Indonesia. The results show four key determinants influencing (constraining) adoption were (1) the primacy of profit-seeking objectives in operational planning and development; (2) the availability of sources (clinker substitutes and alternative energy fuels); (3) the limited access to cash capital; and (4) the complexity in implementing emissions reduction projects. The inquiry also compares determinants in an emerging and developed country to provide a comparative perspective on emissions management in manufacturing. It appears that firms from the industrial sector in emerging economies have investment strategies that are largely characterised by activities that accentuate achieving financial benefits or best value for money or cost savings in a short time frame, or ‘short-termism’. Currently, greenhouse gas emissions management activities tend to be second-preference strategies for firms in emerging economies, at least in the industrial manufacturing sector.

## Introduction

Climate change is a constant backdrop to economic and industrial growth, especially in emerging and highly populated countries^1,2^. Secondary production, which is the manufacturing process that involves converting raw materials into components or goods, is a leading contributor. It emits around one-third of global greenhouse gases (GHGs)^3^. Materials processing operations, such as energy and emission-intensive steel and cement making, create nearly half of the emissions of the manufacturing sector^4,5^.

Hence, active involvement in climate mitigation from such industry is highly needed to prevent more severe environmental and social impacts^2,6^. Nabernegg et al.^2^ have advised that cost-effective mitigation measures are generally available in heavy industries across the globe. However, the use of climate-friendly technologies and practices in emerging economies remains low and has not yet been implemented thoroughly^2,7^. According to Blok et al.^8^ and Littlewood et al.^6^ several factors influence firms’ level of enthusiasm and likelihood of participation. Lack of understanding of the decision to adopt emission reduction potential has resulted in the drivers not being facilitated and the inhibiting elements not being overcome^9^.

Numerous enquiries have outlined reasons behind the decision to adopt climate-friendly practices in heavy and emission-intensive industry. Studies mostly carried out in a developed country context, have identified several factors influencing firms in adopting climate-friendly practices, namely: knowledge capital and cost-saving, followed by environmental regulations, environmental management tools and organizational changes^10^, profit, competitiveness, public pressures and environmental awareness^11^ and stakeholder pressure and increasing competitiveness^12^. In addition, Reddy^13^ has analysed the determinants of environmentally friendly investment with an actor-oriented approach. It resulted in a new systematic classification and description of barriers and drivers to help policymakers evaluate and assess the success of specific interventions.

Other enquiries reveal that business matters (e.g. cost saving and increasing competitive advantage) and stakeholder pressure (e.g. Government regulations and customer demands) are the main drivers that enhance heavy and emission-intensive industry in developed countries’ commitment to climate action, rather than sustainability or social and environmental awareness^6^. These findings are in line with previous research in developed countries which show that government pressure is the main factor driving industry to adopt emission reduction measures^14–16^. Specifically, Schönsleben et al.^17^ found that economic drivers are the key ones in heavy and energy-intensive manufacturing, such as cement making.

Apart from drivers, knowledge of barriers within firms to effective mitigation is also essential^18^. Venmans^9^ argues that overcoming these barriers could yield climate-friendly practices at very low cost. The most significant ones faced by manufacturing sectors in developed countries also vary, such as lack of a robust policy framework, followed by uncertainty about government action and the marketplace^11^, lack of clarity in a mitigation strategy^19^, technical risks^13^ and cost or economic matters^18,20^. Specifically, Venmans^9^ showed that capital availability is the foremost hindrance in heavy industry in developed countries, even if it could offer cost savings. Moreover, Ren^21^ identified other significant barriers faced by emission-intensive companies in developed countries, such as a shortage of staff and time and a lack of prioritisation.

While some researchers have appraised several fields of production at once^9,18^, others are oriented to specific industries and locations, such as the manufacturing sector in developed countries^12,22^, small to medium enterprises^23,24^, and large-scale industries^6,25^. Studies have emphasized that the decision to choose the best and appropriate mitigation measures should be based not only on potential emission reductions and profits, but also on an understanding of the factors that could potentially drive and hinder implementation^26,27^.

From an engineering backdrop, this project probes determinants of mitigation performance in a developing country, via study of a prominent heavy industry. It will first establish a literature review related to low carbon practice. Subsequently, results identify the drivers and barriers to adopting emission reduction measures with formative discussion to follow. The investigation offers discussions and conclusions for future enquiry. Finally, methods, data collection, the theoretical framework, and analytic techniques are described. The investigation offers conclusions and recommendations for future enquiry.

## Results

### Drivers

Research on emission reduction strategies has provided further understanding of the involvement and commitment of businesses to adopt emission reduction measures. Drivers are factors which influence agents to take mitigation measures^25^. Although mitigation is recognised as necessary to climate-friendly operations, motivating factors can differ in each organisation, or even among relevant policymakers^9,28^. From a targeted literature review and interviews conducted with several key actors who are closely related in the development of climate strategies in corporate groups, this study has identified six drivers, now presented in micro (internal to a firm) to macro (external to a firm) order.

#### Internal drivers

##### Financial interest or profit

The most crucial factor influencing the firm in adopting mitigation measures is financial interest, namely, opportunities to make a profit, save cost and/or minimise expenses.

Yes, because we are a business, our primary motivation underlying an action is profit, indeed from the financial side. (R3).

Yes, we just look at which strategy is more efficient in terms of financial expenditure, and with the most significant results, well, that option will be chosen […], and so cost-saving is the main thing. (R4).

Respondents also identified several emission measures recently considered most appropriate, along with the relevant reasons. Thus, the company will be likely to maximize its utilisation of clinker substitute materials and alternative fuels. Besides their operating savings or cost-effectiveness, these moves do not require high capital expenditure.

Why do we choose to use clinker substitutions such as limestone and fly ash? Because they provide cost reductions and are less likely to require capital expenditure. That is what we are trying to pursue, and they also can reduce emissions. (R2).

##### Availability of sources

Since clinker substitutes (fly ash and limestone), and alternative fuels (AFs) (refuse-derived fuel (RDF) and biomass) are widely available near the East Java complex, location alone would increase the likelihood of their adoption. For example:

The potential for profit or saving is the highest priority factor in choosing measures, then the availability of resources. (R2).

The advantage of our cement complex regarding these measures is the availability of many sources, and not far away, Sir (R1).

Participants continued that, apart from its financial attraction, biomass utilisation raised the well-being of the surrounding community, as indicated:

So, the use of sources that are widely available in the vicinity and low price will lead to savings […] well, if we use biomass, though it is currently still not optimal, it will not offer simply savings but also it can make the surrounding community happy since it provides business opportunities for them. (R5).

##### Internal awareness

Growing internal awareness of the importance of business strategy to protect the environment and demonstrate corporate social responsibility is another driver. The firm proves its commitment not through coercion but by consistently maintaining environmental quality.

The company has been aware of environmental issues ... it has been well socialised […] So, we continue what has been achieved related to environmental problems even though there are no rewards and penalties from the Government. (R1).

Previous management had initiated energy activities to maintain environmental quality, such that the company has become a leader in Indonesia in achieving energy management certification.

Energy management itself began around the 1990s; we have a collaborative project with Japan, a kind of energy development arrangement. It aims to utilise technology that is more efficient or lower in energy consumption […] We also have been certified as ISO 50001, the first cement company in Indonesia to achieve it. We have an energy manager and an internal energy audit team who are all certified. So everything is complete and certified, and we are still using the services of external auditors as well. (R5).

#### External drivers

##### Investor awareness

The awareness of external parties, primarily in developed countries, of the importance of protecting the environment has led to investment in climate action. Such an outlook could dispose corporate policymakers in an emerging economy to be more proactive towards low-carbon activities. In addition to the firm’s own consciousness, investor pressure drives it to carry out carbon management programs to improve its image, not least in gaining financial support.

... because we are now a public company, we must be aware of sustainability issues because our investors also pay attention to these things. (R3).

The current composition of company shareholders is State 51% and public 49%. Several projects to reduce emissions have been underway with financial support and knowledge transfer from external or foreign investors. They include the use of biomass from rice husks as AF developed with overseas support under a Clean Development Mechanism (CDM) and a joint implementation agreement to adopt Waste Heat Recovery Power Generation (WHRPG) project to produce electricity.

I was involved in managing the biomass utilization project as an AF under a CDM and succeeded. The Swedish Government bought the emission reductions obtained. (R1).

The WHRPG project in our company was carried out with assistance from Japan. (R5).

#### Government regulation and societal influences

Adherence to stakeholder demands such as official policies issued by the Indonesian Government was cited by the respondents as a driving factor. In 2012, The Ministry of Industry set out a standard (Permenperind No. 12/M-IND/PER/2012) for emission intensity in the cement industry, requiring each company to reduce CO_2_ output by 2–3% each year until 2020 (The extension or renewal of the regulation has not yet been determined). It is also a cornerstone of corporate social responsibility to create ‘environmentally-friendly’ conditions, support national emission reduction programs and become an exemplar for similar industries.

Well, as to government regulations...we are a State-Owned Enterprise so the Indonesian Government automatically regulates us: therefore we are definitely required to comply with its provisions. (R5).

Other stakeholder pressure, such as customer demands for sustainable products, has not yet become a driving factor in emission management.

We have already received the PROPER award and, in the past, we thought that getting this award could increase the competitiveness of our products ... However, it did not have an impact. Our customer base did not care about which products are environmentally friendly. (R3).

PROPER itself is the program for assessing performance ratings developed by the Ministry of Environment in Indonesia to encourage companies to improve their environmental management.

#### Technological change

The final driver influencing mitigation measures is technological change. Nabernegg et al.^2^ indicate that a corporation’s climate strategy can be influenced by the ability to adopt current innovations or technical advances^2^. Furthermore, the authors state that, in general, the latest developments (low carbon technologies) not only offer a reduction in emissions, but can also save production costs and increase competitiveness^2,29^. The company studied has realized the importance of the ability to adapt to technological improvements as evidenced in the following quotations.

Yes, we are actively enhancing our human resource capabilities to keep pace with changes or innovations through training, benchmarks, and self-learning (R1).

Advances in technology are going to be interesting in the future. We have the motivation to learn the best practices for reducing emissions. (R3).

A respondent stressed that adapting to technological change has been undertaken for a long time. The company’s willingness to learn and acknowledge to these changes has prompted it to adopt them to support its mitigation strategy.

Around the 1990s, we began a collaborative project with Japan, a kind of energy development arrangement. It aimed to utilise the latest technology that is more energy-efficient. (R5).

### Barriers

Research shows that, despite a desire to create competitive advantage, not all organisations can immediately access the latest technology due to several constraints^2,26^. Barriers are factors which reduce the chance of adopting or implementing actions^13,27^. Relevant examples uncovered in discussions with the company include limited capital availability, complexity in adoption, lack of support and proper regulations from government, lack of a robust policy framework, and changes in management.

#### Internal barriers

##### Limited capital availability

From interviews, it emerged that a financial issue, namely, low capital availability is certainly a barrier. Some respondents actually mentioned it as the main one. Current market conditions, being oversupplied, can impact company profits, restricting retained earnings to invest in projects that require high costs, especially for a ‘second order’ objective of reducing emissions^21^. In 2020, the company undertook capital expenditure of US$140 million, or around 32% of its operating profit. The sum will be focused on developing downstream products, such as precast and ready-mix concrete, and also on plant maintenance to anticipate increasingly tight competition^30^.

So, you could say, the main barrier is low capital availability [..] With many competitors and extremely competitive cement prices, management prioritises profits and reduces expenses. So, we tend only to maintain the existing production process. (R1).

Even though the firm has a strong motivation to assess best practices, interviewees re-iterated that ‘the lowest cost’ is a major consideration in choosing measures, regardless of whether they have the potential to reduce emissions.

Should we think about how much emission can be reduced before we agree to finance the projects? It is out of the question, especially if it is expensive. Well, we adjust to the demands from Procurement: of course, they are trying to find the cheapest. (R4).

Respondents revealed that adoption of the latest technology would generally require strong capital expenditure and long-term investment, and is difficult or even impossible to implement if there is no support from other parties.

Measures with significant investments make implementation in the company complicated, so it has not been considered at this time. Frankly, if it relies on internal funding from the company and involves high costs, it might not be possible. It requires support, like from the government. (R2).

##### Lack of a clear, corporate framework

Lack of a clear, long-term and robust environmental policy framework can thwart mitigation efforts. This situation, exacerbated by oversupplied markets, can cause corporate policymakers to focus on short-term objectives or short-termism, rather than more enduring aspirations^31^. It can create uncertainty in implementing a company’s blueprint or an emission reduction road map. Thus, a mitigation strategy can be based primarily on the interests of a current policymaker, who could have different expertise, understanding or interpretation from his/her predecessors.

The current policymakers in the firm do not have long-term, only short-term plans, so what they do is solely to focus on projects that produce short-term results... Previously, we had long-term draft mitigation policies. If I am not mistaken, they stretched until 2024. Now, all that is gone due to the new management in the company which mostly does not have expertise and experience in the cement industry. (R5).

Due to policy ambiguity from top management, one respondent argued that the finance department, which has the expertise to assess the short and long term benefits of investment in mitigation projects, is not empowered or consulted to determine proposals which should be supported.

We (the finance department) are aware that the financing of mitigation measures is not spent in one period or year, and the impact or gain can be appraised only after a while. Well, as financial people, we understand that, but we cannot do anything. (R4).

Therefore, respondents look for political will from a higher authority such as the relevant ministries to enact policies which favour adoption of mitigation projects, now and in the future. Their hope is to increase certainty and continuity in carrying out climate action, despite changes in firm management.

It is better to issue an environmental policy regulation … made by a party with higher authority or at ministerial level that can skirt the board of directors to implement road maps. Thus, to change the policy path would require approval by a higher authority, so that, when there is a change in the board of directors, it will not create an arbitrary change of policies or the main direction of the company. (R5).

##### Changes in operational management

Changes in operational management, especially at the strategic or policy-making level, have the potential to obstruct the implementation of corporate climate action, especially when newly-appointed staff, specifically the board of directors, have less environmental awareness and expertise than their more seasoned predecessors. Minh et al.^32^ mention that the leadership’s technical skills or competencies will have a positive impact on the innovative work behaviour of their employees. Changes which occur abruptly will also affect the stability of management and create more pressure to produce results in a short time. Research has shown that longer tenure of top management or a CEO will significantly improve company profitability and market capitalization^31^.

If there are changes in management, they have the potential to inhibit the application of measures that have been developed, and it will impact our productivity. (R3).

More specifically, one executive emphasized that the difficulty of implementing environmental strategy is influenced by the different expertise and interests of the newly appointed directorship.

If there are changes in board of directors, the implementation of environmental strategy can change according to understanding or interpretation of the new management. (R5).

##### Complexity in adoption

**Figure 1:**
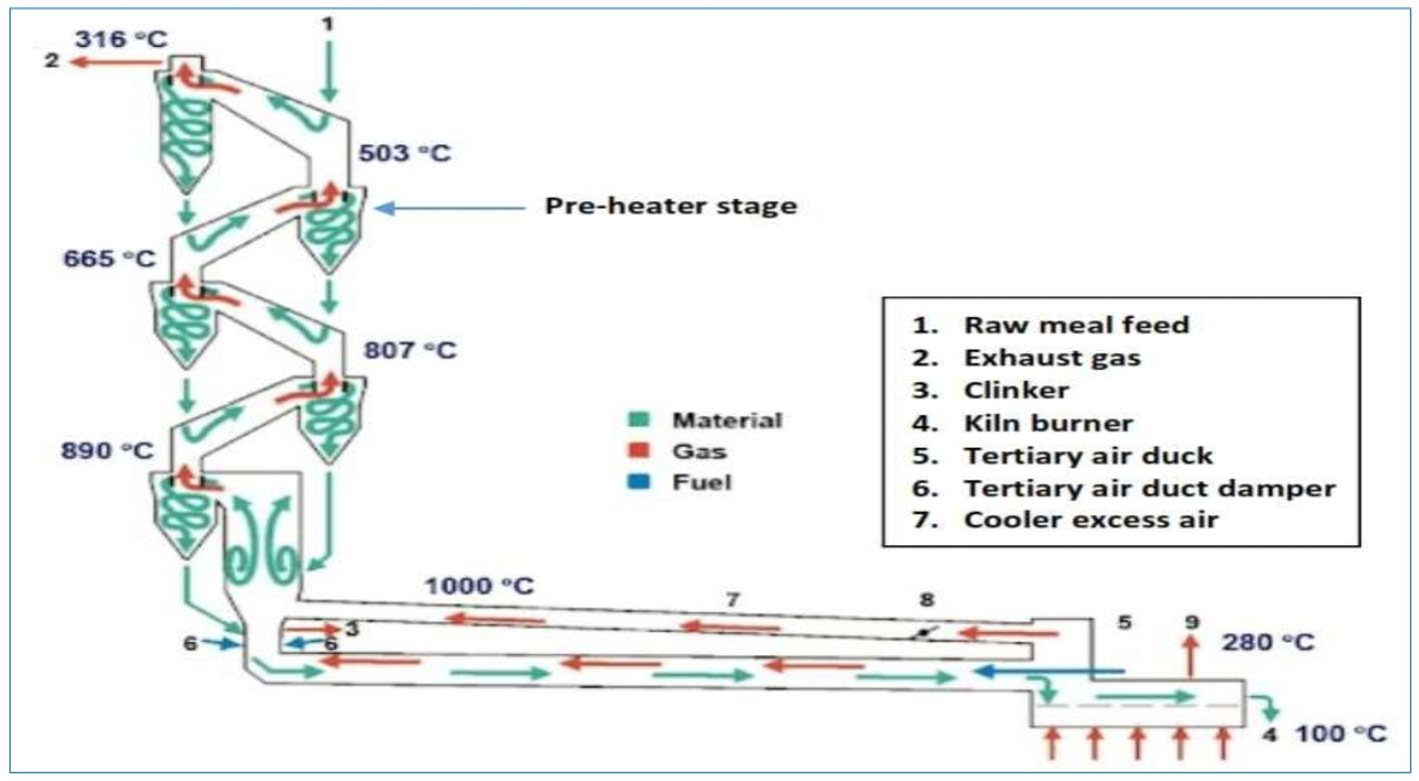
Temperature settings in a dry kiln with five pre-heater stages and pre-calciner^33^.

Complexity in integrating measures is the next barrier. It refers to the difficulty of installation or construction, risk, utilisation, and supervision. For example, the addition of a pre-heater stage in an existing kiln can markedly constrain emissions and energy consumption but entails considerable expertise in construction, adds to risk and would also diminish the waste kiln heat which could otherwise be used as a by-product (Fig. 1). Risk exists because the project will be carried out near other production lines and, physically, must be undertaken at great height (e.g., 100 m). It also requires thorough analysis as regards the suitability of the current structure of the pre-heater to accommodate additional stages. It could potentially require reinforcement of the existing framework in the kiln, or even necessitate entirely new structures and replacement pre-heaters. It will entail ripple costs beyond those of just the incremental phase.

Hmm…the addition of a pre-heating stage, which is revamping of the existing plant, the calculation is very complicated and high risk, but, if it is built (into a new production line) from the beginning, it is easier. So, we have built a new line installed with the five stages of pre-heating kilns. The WHRPG is designed to require a pre-heater exit temperature of at least 400 °C, So, if you add a pre-heating stage, the heat that comes out can go down to around 300 °C, not be enough to meet WHRPG needs. (R2).

Other respondents mentioned that the four existing lines in the cement plant have had a long service life. Lines one to three started operating in 1994, 1997 and 1998, while the fourth came on stream in 2014. The first three were refitted with increased capacity and new technology in 2010. Thus, it would be better to invest in lines which have a greater lifespan, or new installations altogether, as with another line which has recently been built in a location about 100 km from the existing complex.

From a business perspective, adding stages to the current kilns is less attractive because Lines One to Three are around 25 years old. (R3).

Difficulty or uncertainty in obtaining appropriate quality materials from external parties, such as suppliers, has created other problems. The rice husk (fuel) providers and transporters do not always maintain quality control, and can even ‘manipulate’ their product to increase tonnage, but thereby inhibit its utilisation. Supervision is needed not only upon final receipt but from the start of the delivery. Most of the rice growers are local residents who can have limited knowledge of industry. It creates additional disruption or complexity for the company to accelerate the achievement of its mitigation activity targets. The quotation below underscores the realities which can characterise commercial intercourse in an emerging economy.

We also face difficulties with suppliers. We require rice husks with a water content of no more than 20%, but when they send the products, the suppliers or transporters feel that it is still light, so they douse it with water to increase the tonnage. (R3).

#### External barriers

##### Lack of support and proper regulations from the government

Lack of support and proper regulation from the government form barriers to adoption measures. Indonesia has nominated the cement industry as one of the main foci for reducing emissions by establishing a special road map. However, its achievement is likely to be hampered by the absence of a reward/punishment scheme^34^. Policy uncertainty has the potential to retard company operations and sap motivation toward carbon management strategies^35,36^. The lack of seriousness from the government in supporting corporate climate action is expressed as follows:

Some government regulations are not mutually supportive or synchronous, even confusing […] We regularly report our annual environmental performance to the government, Sir, but how do they respond to our report? Until now, we have never been notified. (R1).

The government is more likely to listen to ‘tweeting’ from outsiders, especially environmental activists, who are less knowledgeable about the cement industry than we are. It has disrupted our operations and caused huge losses. (R5).

Regarding inappropriate rules or lack of proper oversight, subjects also mentioned that conflicting regulations caused by inadequate coordination among ministries can interfere with efforts to adopt mitigation measures.

Within the Ministry of Environment itself, there is dissent between the Directorate General of Law Enforcement and the Directorate General of Waste Utilisation. Besides that, the Ministry of Industry requires the use of refuse-derived fuel (RDF) in the cement industry, but the Ministry of Environment limits its usage because of its particulate emissions. All this makes cement companies in Indonesia utilise only a limited amount of RDF. (R1).

Several government regulations appear to inhibit efforts to lower emissions in cement production. First, as indicated, the existing rules regulate the GHG performance of each company based on its respective annual emission discharge, seeking around 2–3% reduction annually. In reality, the emission intensity of cement producers varies, resulting in different target emission threshold values. The challenges faced by each enterprise are likely to be different. Firms should be prompted to adopt processes that are equivalent and create an equitable distribution of business opportunity (levelling the playing field). As an alternative, the government would be better off to set the same target threshold ‘value’ for emission intensity (an emission limit) for all companies. For example, the World Business Council for Sustainable Development has established a viable policy with its emission intensity target value of 0.55 t CO_2_e/t cement by 2030 to support sustainability in the industry around the globe^37^.

Second, the Ministry of Industry’s regulation (Permenperind No.35/M-IND/PER/4/2007) requires that every cement product marketed domestically must comply with provisions of the National Standardization Agency for Indonesia. However, the Portland standard only allows the use of clinker substitutes to 5–15% of the cement composition, although it is possible to increase the rate without compromising product quality.

Third, the Ministry of Environment’s regulation (PP No. 101/2014) regarded fly ash as toxic waste^38^. This stance increased the difficulty in managing permits to use the material. However, at the beginning of 2021, the government issued a Regulation (PP Article 458 (3) C No. 22/2021) which has excluded fly ash from the list of toxic wastes^39^. This new regulation has the potential to enhance cement companies’ involvement in reducing the emissions they produce, especially in maximising its utilization as a clinker substitute.

### The relationship among factors

Numerous studies have recommended further work to promote mitigation measures in heavy industry^9,27,40^. The situation in emerging economies shows how difficulties can combine to compound constraints to cleaner production. To illustrate, in Indonesia, the main barrier to RDF adoption is the need for substantial capital expenditure in sourcing appropriate technology to address the varying quality of municipal solid waste and thereafter reduce annual operating costs. External support is required, such as the provision of tipping fees from the government, as well as funding and knowledge transfer from outside parties^41,42^. A tipping fee applies for units of weight or volume of municipal solid waste accepted for disposal or treatment. The government offers tipping compensation which could become a small income flow for the company. Barriers can be lessened by way of a clear environmental framework within a company’s long-term vision and mission. A viable standpoint would address not only sustainability but also regulate investment criteria for projects which require time to provide a return on outlays and to create savings, particularly in capital-intensive and long-term businesses such as the cement industry^43^.

Beyond such examples, our empirical investigations can be conceptualised via a systematic classification of ‘tactics’ (drivers and barriers) set among relevant contextual influences which affect the adoption of mitigation measures. The influences include ‘technical issues’, ‘company ownership’ (SOE/private) and ‘situation’ (in an emerging/developing country).

‘Technical’ elements in Table 1, as they implicate drivers and barriers, could relate to internal company stances, such as a willingness to restructure or tap new markets, or to external forces including innovations which prompt the firm to adjust to maintain its competitiveness. These opportunities demand working capital which can be in short supply unless immediate financial returns are in the offing. The firm in question also faces constraints due to the lack of awareness and knowledge among suppliers in providing suitable production inputs. Goods offered frequently do not meet specifications, either because of inadequate expertise in product preparation or their being deliberately ‘manipulated’ to reap a sizeable profit, a practice still common in emerging economies.

**Table 1:** The relationship between tactics and contextual influences in an Indonesian cement company.

Tactic		Contextual influence		
		Technical	Company ownership	Situation/location
Driver	Profit	x	xxx	xx
	Availability of resources	xx	x	xxx
	Government regulation	x	xx	xxx
	Internal awareness	xx	xxx	x
	Investor awareness	xx	x	xxx
	Technological change	xxx	xx	x
Barrier	Low capital availability	x	xx	xxx
	Complexity in implementation	xxx	x	xx
	Lack of support and proper regulations from government	x	xx	xxx
	Lack of clear, corporate framework	x	xxx	xx
	Changes in operational management	x	xxx	xx

xxx—Strongly related, xx—Related, x—Limited/weakly related.

In regard to ‘company ownership’ listed in Table 1, State involvement, especially in a developing nation, could putatively increase a firm’s orientation to environmental issues, such as emission mitigation and innovation in environmentally friendly products or services^44,45^. Globally, SOEs produce lower emission levels than private operations^46^. A study by Estrin and Pelletier^47^ argues that privatization alone is no longer the key to generating marked financial benefits in emerging economies, but it can afford an opportunity to increase efficiency and create equality. Olesiński et al.^31^ and Benoit^48^ state that investment and operational decisions in a company are strongly affected by the policy direction, interest and structure of stakeholders. In SOEs, the chances of adopting mitigation avenues depend upon how much the State as a sole or majority shareholder is involved in climate action, rather, than focusing on short-term benefits or a political-economic approach reflecting certain ideologies^35,48,49^. If the State is not prepared for ideological (i.e., market distortion) or fiscal (national budget constraint) reasons to invest in mitigation measures, one route could be to increase the share of private stockholders though, as will be shown, this approach comes with few guarantees of significant action when a market is oversupplied.

### The situation

**Figure 2:**
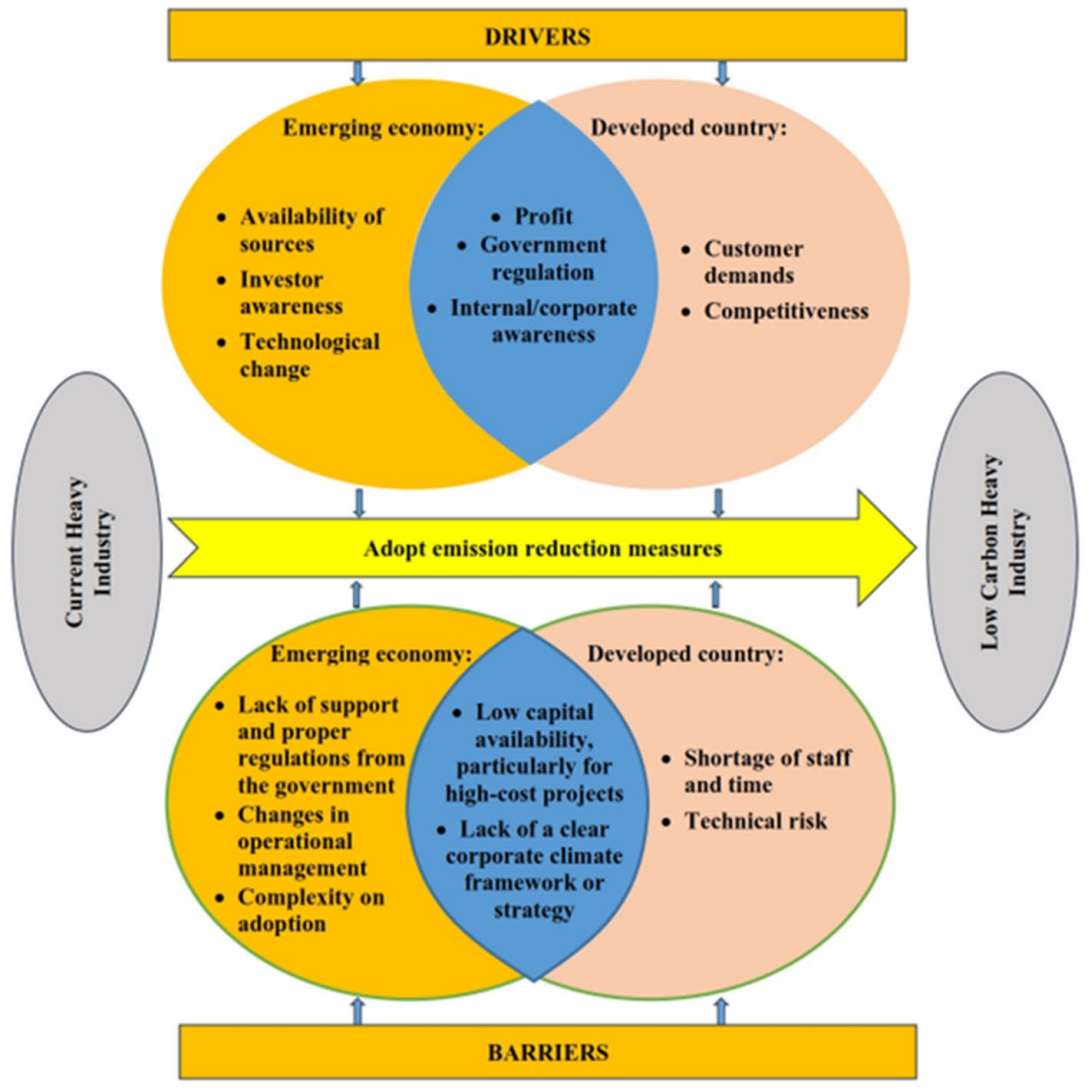
Conceptual diagram depicting factors which influence the decision to adopt emission reduction potential in heavy industry in an emerging economy and developed countries.

The ‘situation/location’—displayed as the most important influence in Table 1—relates to determinants of corporate environmental investment as between emerging and developed economies. In Indonesia, the main driver for transition towards low-carbon operations is the possibility (or otherwise) of profit, followed by the availability of sources or materials (Fig. 2). The case study company has taken up cheaper measures offering quick returns and less challenge in implementation. In developed countries, the main stimulus is stakeholders’ pressure (tight government regulations and market exigencies) and financial imperatives (increasing competitiveness). These nations have better social education to adapt to the green economy, and customers’ environmental interests sway their choice of products^6,19^. In emerging economies such as Indonesia, this sentiment is not yet apparent^50^. Government regulation is indeed one of the driving forces, but not a primary one as it has been in developed countries.

Extrapolating from Fig. 2, both emerging and developed economies can experience low capital availability or priority in adopting measures. Either condition restrains efforts to achieve a low-carbon industry. In the latter nations, difficulties in obtaining financial support generally occur when trying to adopt cost-efficient and technologically complex measures, such as carbon capture and storage (CCS)^21,51^. In the current analysis, the lack of support and appropriate regulation from the government, the complexity of adoption, and changes in management are additional barriers, but are not immediately visible^11,21,52^.

While the Indonesian case study thus bears limited resemblance to experiences in developed nations, what of the second research proposition regarding parallels with other emerging economies such as India and China? These two countries have the largest cement markets in the world but, unlike Indonesia and many other developing counterparts, have binding regulations for business actors to reduce their emissions. Similarities no doubt exist in corporations’ aspiration to reduce their environmental footprint, along with shared interests in compliance and transparency as a manifestation of their ability to adapt to new values. Awareness of stakeholder interests appears to be increasing. Common themes among the barriers to adoption include the shortfalls in financial access and capability to achieve state-of-the-art abatement measures. These problems are not about to disappear in the short term and could require external aid or investment to overcome^26,52^.

## Conclusion

This study has deepened knowledge of the determinants which underlie a company’s quest to lower emissions in the framework of an emerging economy. A unique case study because it was conducted in a country with oversupply market. It has put forward a taxonomy of drivers and barriers around three contextual influences, namely technical issues, company ownership and situation. Several findings merit attention. First, profit and availability of sources or materials are the most important drivers in carbon management, followed by government regulation, internal and investor awareness, and technological change. The primary barrier is low capital availability, attended by complexity in implementation, and insufficient support and proper regulation from government, lack of a robust policy framework, and changes in management.

Second, mitigation measures can involve operating adjustments such as the scaling up of existing procedures (i.e., the use of clinker substitutes and alternative fuels) which are likely to be adopted before strategies involving substantial capital expenditure (e.g., adoption of the best available technology). Measures are generally influenced by their risks, their level of effectiveness, cost, capability, and uncertainty about the implementation process.

Third, barriers tend to be intertwined and mutually reinforcing, a situation which can account for the general inactivity toward climate action and a wavering focus on emission reductions. Operational investment in the over-supplied Indonesian cement market regularly reflects a ‘best value for money’ approach. The case study firm will carry out a carbon management program to the extent that it promises to save costs or raise profits. Rather than significant capital expenditure, it is inclined toward cheaper operating measures.

Fourth, ‘situation’ is the most important contextual influence related to a firm’s environmental investment. Heavy industry in emerging economies will generally face more challenges that demand attention in adopting mitigation projects than will similar firms in developed countries. As global concern to tackle climate change grows, firms will need to consider emission measures to respond to increasingly stringent environmental regulations and international and market pressures. They should refer to best practices or aim to mimic those in advanced nations, for example considering AFs alongside advanced technology, such as five to six-stage kiln pre-heating. Government ‘carrot’ incentives might initially have some purchase in the existing situation but, in the longer term, if emerging economies are going to meet their 2030 United Nations Nationally Determined Contributions (NDCs), governments might have to regulate domestic and foreign-invested firms in a heavier-handed way. The NDC itself is a national plan highlighting climate actions, including targets, policies and measures governments can implement in response to global efforts to address climate change. In accordance with the Paris Agreement Article 4, Paragraph 2, it requires each country or party to the UNFCCC to prepare, communicate and monitor successive steps which it intends to achieve. The NDC thus involves pledges to mitigate GHG emissions.

From the inquiry, several key management insights and wider applications can be drawn apropos environmental management in heavy industries in emerging economies. Adopting mitigation measures might not be undertaken immediately because of concerns that they will disrupt production. It is critical to have senior officers with capability and responsibility for climate action, such as an energy and environmental manager. They should first assess and determine the company’s climate posture before aligning it with business strategy^53^. They also need close access to the board of directors to assist leaders with diverse backgrounds, co-opting those who are less familiar with relevant issues. Despite their significant size and technical competences, major companies in developing countries still need to cooperate with international parties or donors to acquire support, funding and knowledge transfer for complex investment projects.

Regarding the three research ‘propositions’ articulated in the ‘Materials and methods’ section, the Indonesian example cannot be reliably matched either with that of firms in other advanced nations or emerging economies. Viewed differentially, attention should fall upon on the nation’s excess cement production capacity. Together with imports, it left the market oversupplied by 45 Mt in 2019^54^, underlining the highly competitive conditions alluded to by interviewees in this project. Within the marketing mix, price and product (development) seem more important facets of industry conduct than the non-price aspects of promotion, place and people. The market structures likely account for the short-termism noted by respondents, the intense focus on costs and profits, and the constrained response to apparently second-order desiderata such as climate change mitigation. This situation is unlikely to alter, especially if foreign entrants simply maintain or recycle (i.e., ship in) elderly plant. To make any real difference, they would need to cut through the market constraints by installing cost-efficient, up-to-date technology (e.g., six-kiln lines), though this move implies capital intensity which would reduce the demand for labour in situations of abundant supply. It might also be risky, were other companies still importing low-priced cement products. This Gordian knot illustrates some of the operating traps faced by emerging countries in achieving world-class emissions abatement.

Given the critical fact of its saturated, open market, Indonesia therefore appears to depart from both the developed and emerging country cement markets posited as analogues. Reflection around the existence of State-owned enterprise begs a further question: what would happen if the nation’s cement companies were fully privately-owned, either domestically, or through foreign direct investment, or through the two means in combination? Privatisation is generally undertaken to increase management efficiency so that a firm can compete optimally and quickly adapt to emerging values^55,56^. Private companies tend to have more impact on national economic growth^57,58^. However, in fact, leading foreign corporations operating in emerging economies cannot automatically be assumed to have the same environmental performance as those in their homeland or in other developed countries. Take, for instance, Heidelberg, a global cement giant. The direct emission intensity of its plants operating in developed countries has reached around 0.5 t CO_2_e/t cement, but those in Indonesia still record 0.7 t CO_2_e/t, even higher than the state-owned operator^59,60^. Privatisation by itself seems to offer no immediate answers to the abatement problems canvassed in this account. Thus, Wagner et al.^36^ support the presence of clear and robust environmental regulations to improve their environmental performance and achieve more efficient and cleaner production processes.

Acknowledging the importance of tackling climate change, the application of low-carbon technologies is indispensable. Innovations and the latest technology to achieve cleaner production generally come from developed countries. Comparatively, the costs of new technologies in most energy-intensive industries are lower in emerging economies, but their adoption there is still limited^2^.

In promoting investment for latest innovation or technology, developing economies face constraints, such as low awareness, lack of policy that provides incentives to investment, and limited internal capacity to adopt large-scale projects or technologies that require high cost and technical capabilities^61,62^. However, Si et al.^63^ have stated that the fast economic growth in several such nations has opened opportunities to catch up with advanced countries. As to narrowing the innovation and technological gaps, it is essential to investigate the determinants that drive and limit emerging economies in adopting new technologies so that they can also engage, take advantage of, and develop these advances^63,64^.

Governments can assist by eliminating energy subsidies, setting large energy-saving targets, encouraging research and development into clean technologies, and providing incentives to boost investment in cleaner production or technology^34,65,66^. Companies should also be urged to take advantage of agreements between countries to adopt measures that require significant cost and technical capability. Developed lands can invest in mitigation projects or joint implementation (JI) projects and can meet their domestic targets by purchasing emission reduction units from developing nations or via the clean development mechanism (CDM).

For researchers, practitioners and policymakers, future investigation of corporate climate engagement in the global South could be furthered through: (1) qualitative methods supported by technical details and informed interview techniques which can be productive assuming that appropriate respondents are selected^67^; (2) further exploration of company boards, the largest shareholders and SOE authorities to elaborate the view presented here; (3) academic and regulatory liaison with industry to overcome barriers and to produce better design and implementation of policies; and (4) continued exploration in heavy industry in emerging countries to augment the literature on determinants and the possibility of generalising results.

## Materials and methods

### Ethical note

This study was conducted in accordance with the ethical approval of the School of Earth and Environmental (SEES)—The University of Queensland, Australia ethics committee for human subjects (Permission Number: 201804-07) which complies with the Australian’s National Statement on Ethical Conduct in Human Research and the associated university regulations. All methods were effectuated according to the relevant guidelines and regulations and all participants were informed and gave written consent that they voluntarily participated in the study.

### Theoretical framework

Studies carried out in developed^68,69^ and emerging countries^70^ in key heavy industries (i.e., cement^68^, steel^71^, and energy^72^) and including companies of different size^69^ have shown various drivers and barriers. The strand in developed countries has identified drivers which support businesses adopting climate-friendly practices. They include stakeholder pressure^15,21^ (e.g., government regulations and customer demands), business interests (e.g. cost saving and increasing competitive advantage)^6,9^, followed by corporate awareness^12,21^. Related to these matters, Littlewood et al.^6^ identified in Europe’s high-emissions industry that personal motivation to engage in sustainable activity toward climate change does not positively influence *corporate* commitment to action. The main barriers are insufficiency of capital or a low priority for investment projects with significant cost^9,52^, lack of a robust environmental strategy^11,52,73^, shortages of staff and time^21^, and technical risk^13,74^.

In emerging economies, the background remains patchy and unclear, since effective mitigation technologies and practices are not extensively employed^2,7^. Lagging appreciation or study of the determinants underlying management’s decision to adopt potential emission reduction measures has resulted in the drivers not being facilitated and inhibiting elements (i.e. barriers) not being overcome^9,75^. In countries such as China and India, government pressure is not a key issue, and nor does it exercise a major impact on corporate environmental performance, since legislation tends to be flexible or voluntary^15,76^. Additionally, Zhang et al.^15^ and Singh et al.^76^ found that larger scale significantly impacts an enterprise’s proactivity in environmental management.

The main influences are quite varied, ranging from international and external suasion^77^, market benefits^19^, organizational capability^19,78^, and internal and stakeholder sentiment^76,77^. The barriers consist of shortfalls in financial availability, awareness and capability^19,26^. Firm size, location, value and export orientation have additionally been mentioned by authors^79^. Moreover, Venmans^9^ undertook a study using neo-classical economic theory and behavioural economics to identify how top management in energy intensive companies make decisions to adopt mitigation measures such as energy efficiency. It found that the most significant barriers are strict capital budgeting rules and lack of information and knowledge. It also mentioned that the direction of internal capital budgeting and the study of technical feasibility and profitability are relevant to understanding the efficiency gap.

Finally, according to Böttcher and Müller^12^ and Si et al.^63^, the differences between drivers for, and barriers against, process innovation significantly relate to three factors, namely location (emerging and developed countries), company characteristics (i.e., class of industry, size, and ownership), and type of innovations. On this basis, the present research needs to define control variables to gain a more comprehensive understanding in emerging economies of management decisions to adopt environmental innovations to achieve a distinct advantage^63,80^.

**Figure 3:**
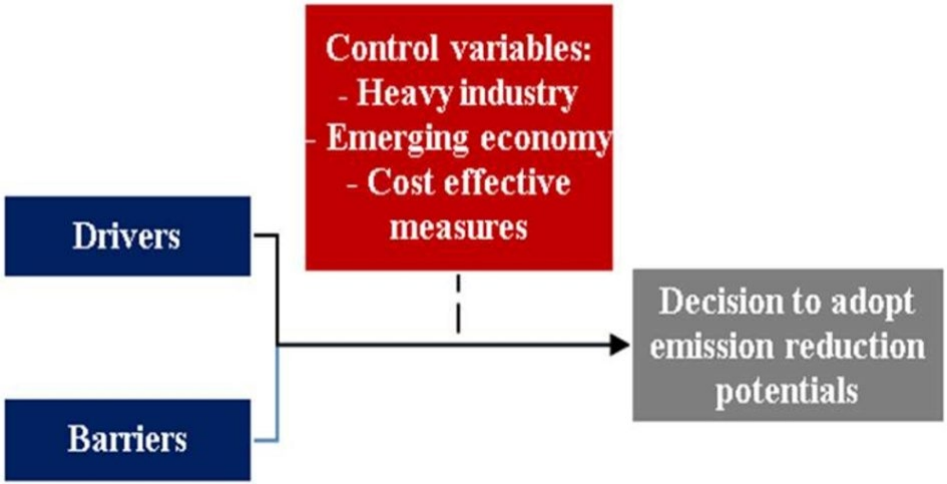
Theoretical framework of factors that influence the decision to adopt emission reduction measures in heavy industry in an emerging economy.

We formulate a conceptual framework to probe certain emission reduction measures (Fig. 3). It parallels the drivers and barriers which influence the control variables facing management. This tripartite combination lies behind a decision (whether) to adopt emission reduction measures.

### Research propositions

Earlier, we have cited influences supporting and inhibiting the uptake of climate-friendly technology in heavy industries in developed countries, alongside a lesser base of evidence applying to emerging nations. Several differences are apparent. Compared with developed countries, the latter tend to have limitations related to a lack of awareness and capability. Besides, the role of stakeholders (including government and customers) in such economies is still not directed to encourage the commitment of business actors to climate action. From this springboard, three research ‘propositions’ inform the current project. First, given the level of foreign investment in the Indonesian cement market, it could be that the environmental response in the case study firm will resemble that of companies in advanced nations. Second, because of market and technological constraints, the firm might follow the means of mitigation apparent in the emerging economies (China and India) just examined. A third (idiographic) possibility is that the firm could post an individual course determined by its own domestic situation. Whatever the outcome, empirical investigation could produce a worthwhile advance in theorising, setting up a platform for further enquiry.

### Participation and procedure

Following our previous technical research^67^, the project employs a phenomenological approach in conducting in-depth, first hand discussions with several senior company executives^81–83^. Paralleling the work of Si et al.^63^, five senior participants, all male, were interviewed, each of whom had core responsibilities for, and/or involvement in, the development of climate strategies^84,85^. The number of respondents can be considered adequate if the information already obtained tends to be repeated from one to the next^85,86^. Four interviewees work in technical and production activities and the fifth is from the finance department of the case study firm. Based around a structured questionnaire featuring open-ended items, meetings were occurred between September and October 2019, both at the production complex in East Java and at headquarters in Jakarta, the Indonesian capital.

At the beginning of the exchange, the interviewer reported the results of previous carbon analyses (emission inventories and abatement costs) carried out in the company^67^. During proceedings, respondents addressed four primary domains: (1) explaining: characteristics, objectives, and benefits of mitigation opportunities that have been taken up previously in the complex; (2) understanding: the results of carbon analyses which have been carried out (inventory and marginal abatement cost (MAC)) in regard to the potential and costs of emission reductions from measures developed; (3) identifying: the drivers and barriers involved in choosing among the mitigation actions available; and (4) gauging: the importance of reducing emissions through implementing effective reduction measures.

### Case study setting

Indonesia provides an ideal setting for the current investigation. It is economically the 22nd, and demographically the fourth largest country in the world. It the fifth largest of the emerging nations and, with a GDP of $US1.9 trillion, the frontrunner in southeast Asia. Sustaining annual GDP growth rates which averaged 5.1% over the last 20 years^87^, it features strong demand for basic nation-building resources. Correspondingly, its cement industry, the focus of the current study, is ranked fourth largest among those of the emerging economies and sixth in the world^88^. Since per capita cement consumption remains low, Indonesia attracts new foreign entrants. While they might have missed a first-mover advantage, they can still secure a foothold in a market with long-term expansion prospects given its substantial infrastructure and construction needs. Yet, as now explained, this market features certain structural peculiarities which can affect corporate conduct and performance.

**Figure 4:**
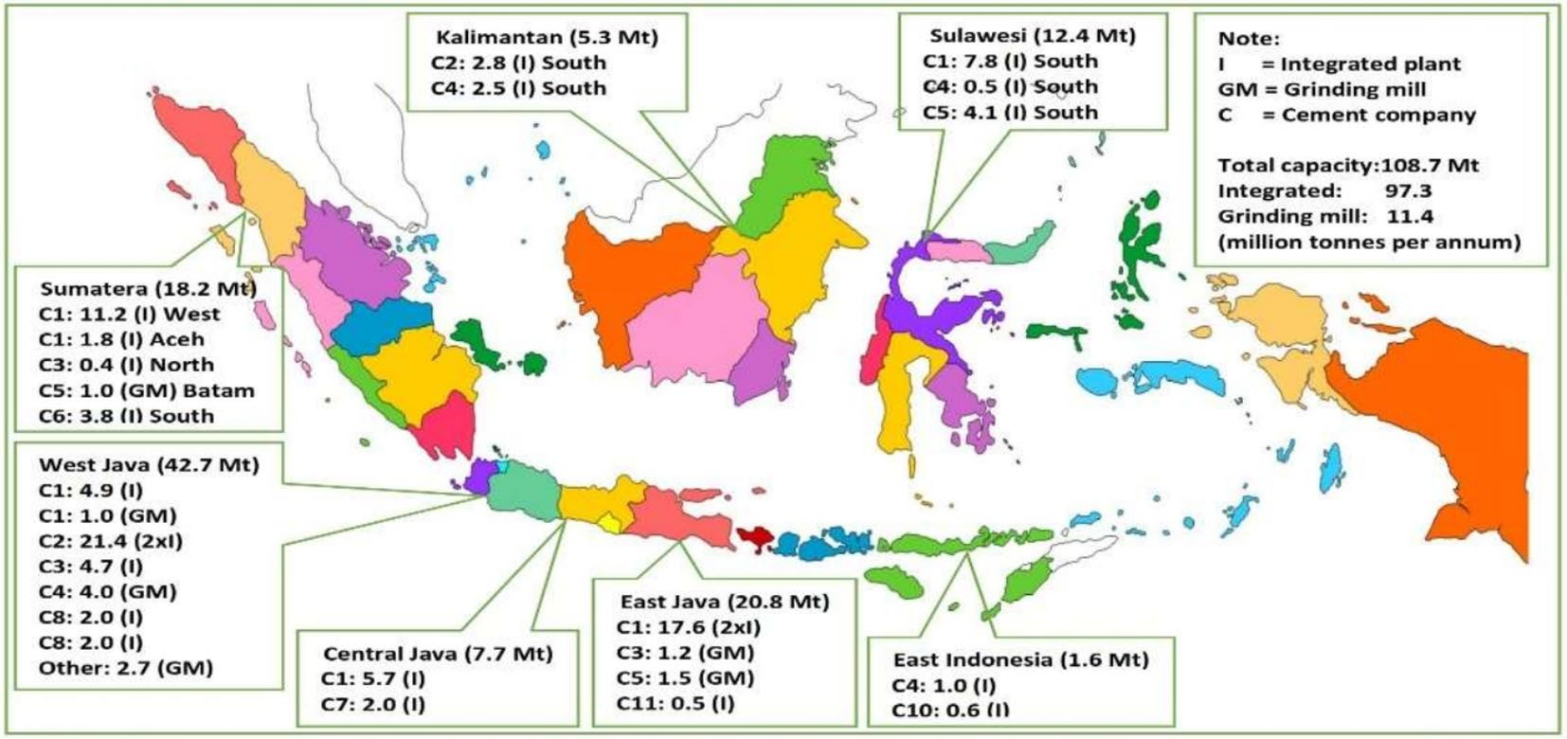
Market structure, cement supply, Indonesia, 2018^89,91^.

**Figure 5:**
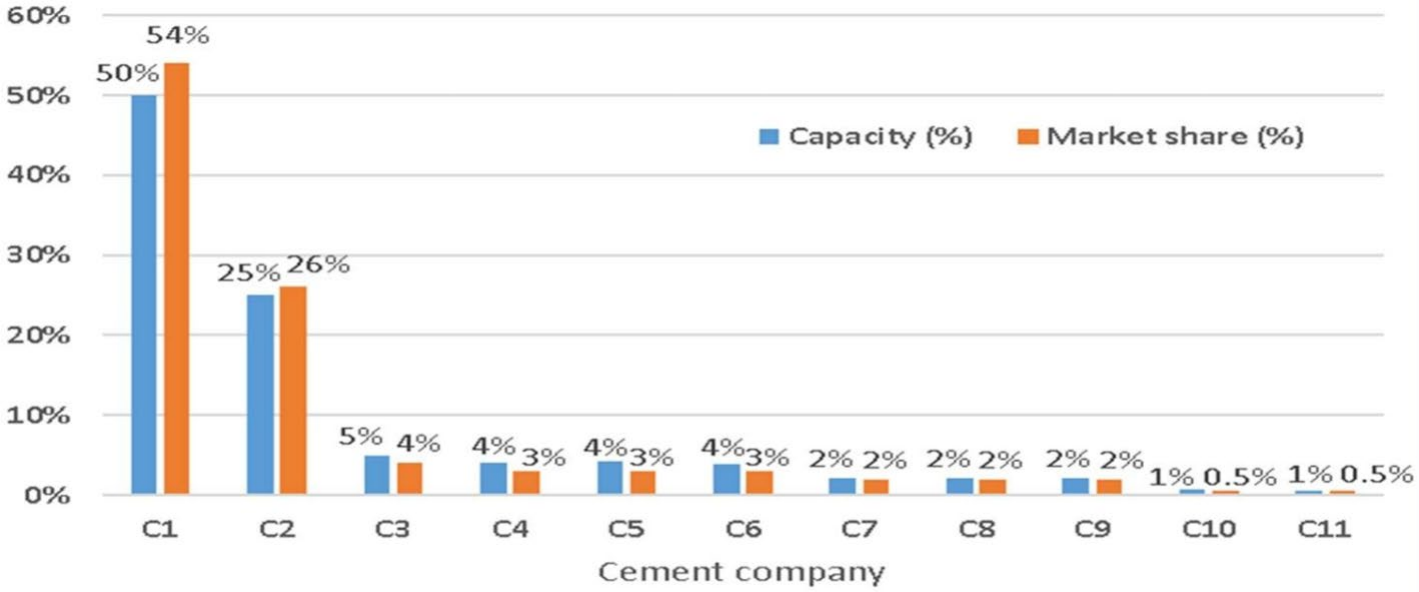
Geography of cement supply, Indonesia, 2018^89,91^.

First, since 2017, installed cement capacity has exceeded domestic demand by around 35 Mt per year^89^. Currently, 80% of the capacity in Indonesia is dominated by only two companies (Figs. 4, 5)^89^. The case study was conducted at the leading group, holding around 50% of national production capacity. It controls nearly 55% of the national market, with a utilization rate of its installed capacity over 80%^30^. It also undertakes cement exports to compensate for domestic market conditions, which are very competitive due to continuing oversupply^90^. Indeed, cement is still being imported, especially by several new players. The practice is attended by periodic allegations of ‘dumping’ or ‘predatory pricing’ usually designed either to reinforce a niche position or to waylay potential competitors from entering the same market^54^.

Second, across the globe, many companies within heavy industry, being energy and emissions-intensive, are partly or fully owned by the State. In Indonesia, the existence of such State-owned enterprises (SOEs) reflects the highest constitutional mandate, the 1945 Constitution of the Republic, Article 33. The Article requires that the economy be built on the principle of kinship. Production activities which control the lives of many people, including land, water and natural resources, are owned and managed by the State for the welfare of the people. Three of the 11 companies in the domestic cement industry feature this type of ownership which has the potential to influence behaviour regarding emission mitigation.

The case study is directed to one of these firms. Previous marginal abatement cost research in the largest (four-line) cement plant owned by this group identified six cost-effective GHG mitigation measures^67^ (Table 2). The most significant is offered by fly ash (FA) as a clinker substitute, saving 1,026,890 t CO_2_e/year at cost of -US$9.15/t CO_2_e reduction. The next is the use of up to 10% of limestone (LS) as a clinker substitute (981,296 t CO_2_e/year, US$11.07/t CO_2_e). Biomass utilisation could replace up to 25% of coal consumption (936,046 t CO_2_e/year, US$17.68/t CO_2_e); adding a pre-heater stage in the kilns (224,651 t CO_2_e/year, US$7.44/t CO_2_e); WHRPG (147,688 t CO_2_e/year, US$10.12/t CO_2_e); and use of RDF of up to 130 t/day as an alternative fuel (97,948 t CO_2_e/year, US$15.38/t CO_2_e). Four of these measures require up-front capital costs. Strong investment is needed for WHRP (US$60 million), likewise the addition of a pre-heater stage in kilns (US$26.5 million). Plans to enable the combustion of biomass and RDF would respectively entail set-up costs of US$3.1 million and US$4.3 million.

**Table 2:** Marginal abatement cost of mitigation measures^67^.

Abatement measures (Listed in US$ savings order)	Capital cost, year 0 (US$ million)	Cash flow (US$ million/year)	NPV (i = 0.1, n = 10) (US$ million)	Total abatement (t CO_2_e/year)	Abatement saving (US$/t CO_2_e)
Biomass (substitute for 25% of coal)	3.143	27.448	165.511	936,046	− 17.68
RDF	4.286	3.149	15.061	97,948	− 15.38
LS (clinker substitute by 10%)	–	17.684	108.659	981,296	− 11.07
WHRPG	60.000	12.198	14.952	147,688	− 10.12
FA (clinker substitute)	–	15.287	93.933	1,026,890	− 9.15
Add one pre-heater stage in kiln	26.530	7.037	16.710	224,651	− 7.44
Total	93.959	82.803	414.826	3,414,519	− 70.84

## Data Availability

The datasets generated and/or analysed during the current study are not publicly available due to restrictions or confidentiality (e.g., their containing information that could compromise the privacy of research participants and to avoid possible conflicts with other parties) but are available from the corresponding author on reasonable request.
